# Investigation of the Technological Possibility of Manufacturing Volumetric Shaped Ductile Cast Iron Products in Open Dies

**DOI:** 10.3390/ma16010274

**Published:** 2022-12-28

**Authors:** Vladimir Galkin, Andrey Kurkin, Gennady Gavrilov, Ilya Kulikov, Evgeny Bazhenov

**Affiliations:** 1 Department of Mechanical Engineering Technological Complexes, Nizhny Novgorod State Technical University n.a. R.E. Alekseev, 603950 Nizhny Novgorod, Russia; 2 Department of Applied Mathematics, Nizhny Novgorod State Technical University n.a. R.E. Alekseev, 603950 Nizhny Novgorod, Russia; 3 Department of Materials Science, Materials Technology and Heat Treatment of Metals, Nizhny Novgorod State Technical University n.a. R.E. Alekseev, 603950 Nizhny Novgorod, Russia

**Keywords:** forging in open die, ductile cast iron, ultimate plasticity, analysis of the stress–strain state of the material, microstructural studies

## Abstract

Information about the technological possibility of stamping in open dies, round-shaped forgings made of ductile cast iron, is outlined herein. Cast iron’s propensity for plastic deformation under complex loading conditions is analyzed from the standpoint of the morphology of graphite inclusions, depending on the degree and mechanical scheme of deformation. The research methodology included: the choice of a brand of cast iron with spherical graphite and the technological process of its deformation; mathematical modeling of the deformation process using the DEFORM–3D software package; stamping of an experimental batch of forgings; and microstructural studies of the forging material, together with the data of its stress–strain state and the direction of flow of the material. Under the conditions of comprehensive compression of the workpiece material at the beginning of the deformation process, lateral pressure was created from the tool walls. It was carried out by selecting the size and shape of the initial blank by mathematical modeling. When analyzing the morphology of graphite inclusions, the stress state scheme was determined as the main influencing factor. The greatest change in the shape and size of graphite inclusions of cast iron, including their crushing, corresponds to the condition of the disappearance of all-round compression. When stamping in open dies, this occurs in the area where the material exits from the stamp engraving into the flash gutter. In the process of forming, cast iron showed the possibility of deformation in the deformation intensity index ε_i_ = 2.5.

## 1. Introduction

In mechanical engineering, one of the important practical tasks is the manufacture of products from hard-to-deform metal materials, one of which is cast iron. As a structural material, grey cast iron contains, in the microstructure, graphite, which is widely used for the manufacture of various parts. Compared with steels, it has a number of advantages: it works well on friction, is wear-resistant, and dampens vibration. However, cast iron is inferior in strength due to the low level of viscosity and fracture resistance, which is due, on the one hand, to the presence of graphite particles, and on the other, to defects in the structure of the cast metal [1,2,3,4,5]. The first studies on plastic deformation of cast iron containing graphite in its structure date back to the end of the 1940s. The starting point of these studies was the experiments of T. Karman, Becker [4,5] and the fundamental works of P.V. Bridgman (1947) [6,7,8], the results of which showed that even brittle materials, such as marble and red sandstone, can be plastically deformed under conditions of uneven all-round compression. In practice, the scheme of uneven triaxial compression was implemented in the 1950s in the technological processes of extrusion, pressing, and volumetric stamping. As concrete examples of the manufacture of cast iron blanks, one can cite: hot rolling of a sheet to a thickness of 1.5–2.1 mm from a rectangular-shaped slouch in several passes and the production of pipes by pressing, stitching, and rolling, obtaining grinding bodies (balls) by the process of cross rolling [7]. The mechanical properties of cast iron depend on the shape of graphite [1]. Analysis of the deformability of cast irons with different graphite shapes has shown that as graphite inclusions approach the spherical shape, the ductility of cast iron increases significantly. The spheroidal shape of graphite does not have a strong incising effect on the metal base, as a result of which a stress concentration occurs to a lesser extent around graphite spheroids. For comparison, the value of elongation in tensile tests for cast iron with lamellar graphite is 0.2–0.5%, and with spherical graphite, it is 10–12%. Ductile cast iron has a high ratio of conditional yield strength σ_0.2_ to the temporary tensile resistance σ*_b_*, which is 0.6–0.7, which allows it to be used as a structural material along with steel (steel: 0.5–0.6) [9]. The second decade of our century has been marked by a large number of studies in the field of hot plastic deformation of ductile cast iron, in particular, those carried out by scientists of the Belarusian school of deformation [10,11,12]. The research direction concerned changes in the structure, in particular, the shape of graphite particles, under various deformation schemes and their influence on mechanical properties. The main results of the research were formulated into the following conclusions:

- The structural, mechanical, and physical properties of cast iron are determined by the shape of graphite inclusions, the changes in which are determined by the deformation of the metal base (matrix) of cast iron [13,14].
- Depending on the mechanical deformation scheme implemented, graphite inclusions of cast iron acquire a different shape: a disc-shaped shape under precipitation conditions, an elongated shape during extrusion [11].
- Studies of plastic deformation of cast iron under conditions of complex loading are rationally carried out using technological samples in which deformation schemes are implemented in relation to the manufacture of a certain class of products [15].
- The plastic flow of brittle graphite inclusions inside a metal matrix must be performed under conditions of uneven all-round compression; however, it is possible to use deformation schemes with the presence of tensile stresses [16,17]. As a confirmation of the use of deformation schemes that do not fully correspond to comprehensive compression, examples of the developed processes are given: backward extrusion in one pass of the “electric drill chuck body” part, hot transverse three-roll rolling of parts such as rods and shafts, and cold surface rolling with rollers [18,19].

When analyzing the developed processes of plastic deformation of cast iron to date, the practical interest in the manufacture of volumetric shaped products using deformation technological schemes that differ from the schemes of the implemented processes should be noted. In particular, this applies to the process of hot volumetric stamping in open dies, which makes it possible to produce products of various shapes. The shaping of the material in open dies is characterized by uneven all-round compression at an average total pressure and incomplete lateral pressure of the metal on the rigid walls of the tool. During deformation, part of the metal is displaced from the engraving in the form of a technological burr, which causes local free broadening of the metal in the area of the exit into the burr groove, creating additional tensile stresses that contribute to destruction. The structural features of the deformation focus and the periods of its change during stamping in open dies are described in detail in the works of M.V. Storozhev, S.I. Gubkin, E. I. Semenov, and other scientists [20,21]. To date, hot stamping of volumetric shaped ductile cast iron products in open dies has no practical implementation, which allows us to conclude that this research topic is relevant to determine its technological capabilities for the manufacture of specific classes of products. The mechanical properties of the material depend on its metallurgical and structural condition. In the process of plastic deformation of the material, the defect and structure parameters change: grain size, morphology of the structure, residual micro, and macro stresses. Therefore, the problem of determining the technological feasibility of manufacturing volumetric shaped ductile cast iron products in open dies requires solving theoretical issues to establish patterns of changes in its structural state depending on the deformation conditions, which was the purpose of this work.

## 2. Materials and Methods

The solution to this problem involves the application of a comprehensive research methodology, which includes: mathematical modeling of the process, with the determination of the optimal dimensions of the workpiece and the assessment of the stress–strain state and the nature of the flow of the material in its volume during deformation; stamping of the experimental batch of forgings; and structural studies of the deformed material.

Ductile cast iron in a ferrite–perlite matrix with the following characteristics was selected as the material under study:

- The strength characteristics are hardness 170–180 HB, which corresponds to the ultimate strength 490–530 MPa.
- The permissible degree of deformation in hot precipitation is ε_h_ = 12%.
- The chemical composition of cast iron is shown in Table 1.

In order to clarify the ductility parameters of cast iron used for deformation, tests were carried out on the hot sediment of turned cylindrical samples with a diameter of 30 mm and a height of 60 mm from the batch of material used. The samples were heated in the furnace to a temperature of 1025–1050 °C and deformed by precipitation on the press with degrees of deformation of 10, 15, and 20%. In order to avoid contact with cold strikers, the deflection was carried out between heated plates. The results obtained showed that the cast iron used for testing has a ductility with a degree of deformation at a hot draft of ε_h_ = 20%. It was also noted that the cooling of the deformed material must be carried out in the furnace at a speed of 100 °C per hour [22]. As a technological test, the process of hot volumetric forging of a round-shaped “flange” part in an open die on a crank hot-stamping press (JSC “Tjazhmekhpress”, Voronezh, Russia) was chosen. In the manufacture of forgings, a technological scheme was chosen for deforming a blank piece of a round shape into an end face without the use of a precipitation operation.

Mathematical modeling was carried out using the DEFORM–3D software package (SFTC, Columbus, OH, USA, Ver. 6.0).

The optimal dimensions of the initial workpiece were determined based on the possibility of creating conditions of all-round uneven compression in it during deformation. According to the modeling results, the dimensions of the workpiece were selected: height 30 mm and diameter 56 mm, which is 4 mm less than the internal size of the flange section of the working cavity of the stamp.

**Figure 1:**
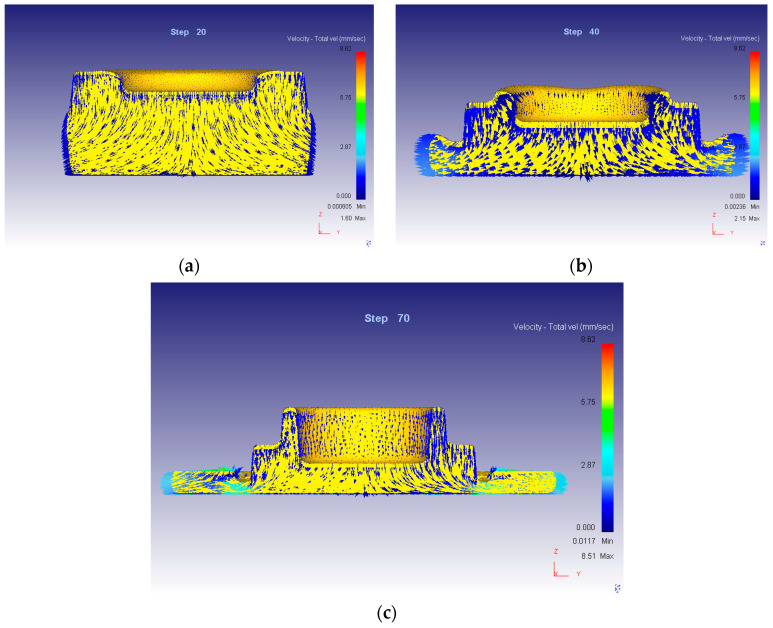
Schemes of the deformed state of the workpiece material: beginning of the process (**a**), intermediate state (**b**), end of the process (**c**).

**Figure 2:**
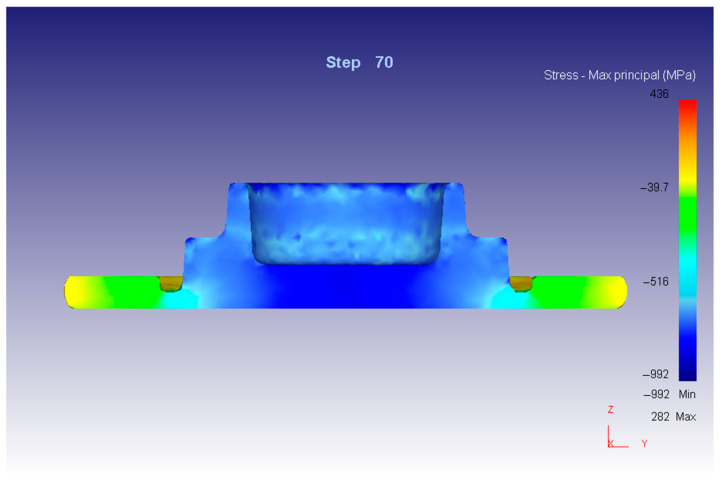
Diagram of the stressed state of the workpiece material at the end of the stamping process.

Evaluation of the stress–strain state in the volume of the product was carried out in areas corresponding to microstructural studies. The schemes of the deformed state of the workpiece material are shown in Figure 1. The diagram of the stressed state of the workpiece material during the stamping process is shown in Figure 2.

**Figure 3:**
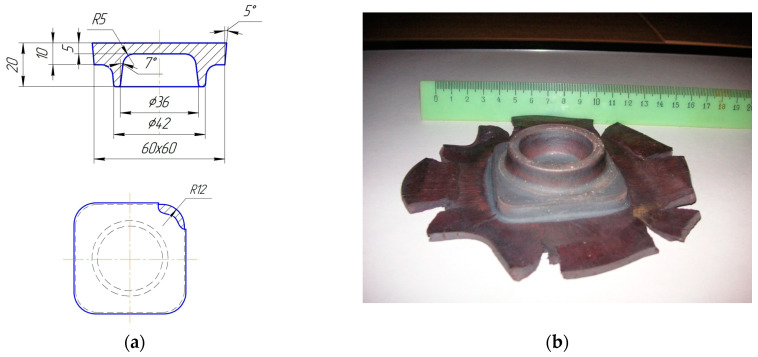
Sketch (**a**) and photo (**b**) of the flange forging.

Stamping was carried out on a crank hot-stamping press with a force of 10 kN. The ground workpieces were heated in an electric furnace at a temperature of 1025–1050 °C for one hour. A sketch (a) and a photograph (b) of the stamped forging of the “flange” part are shown in Figure 3. There are no surface defects in the form of cracks on the forging surface, which indicates that the dimensions of the initial workpiece are correctly determined. After stamping, the forgings were cooled in a furnace of 900 °C and a reduction of 100 °C per hour.

**Figure 4:**
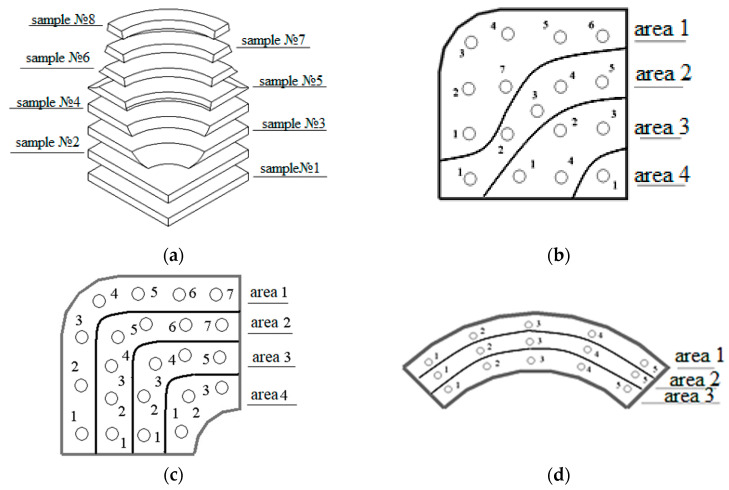
Diagram of cutting the fourth part of the flange forging (**a**) into layered samples; designation of research areas on sample №1 (**b**); sample №3 (**c**) and sample №7 (**d**).

Microstructural analysis was carried out on samples, the cutting scheme of which, from the fourth part of the flange’s forging, is shown in Figure 4a. The measurement of the size and shape of graphite grains was carried out on layer-by-layer and end-face sections of the samples. Zones of the microstructural studies were selected on the planes of layered samples, in which the values of deformation and stress in the intensity indicator were determined using mathematical modeling. For samples №1, №3, and №7, they are given in Table 2 and Table 3.

## 3. Results and Discussion

One feature of the shaping of the initial blank in an open die is the fact that individual sections have different directions of metal flow due to the implementation of various mechanical deformation schemes, which forms a multidirectional structure of graphite particles.

The material of the initial blank is initially deformed in the central zone under conditions of reverse extrusion (Figure 1a). The stressed state is characterized by a comprehensive compression scheme, which is provided by an active force in the direction of precipitation and the reaction of adjacent sections of the deformable workpiece in the radial direction to its axis. The stress state scheme does not change during the entire deformation process; the maximum stress at the end of the process has a value of 239–256 MPa. The flow of metal occurs in both directions from the center in the radial direction (Figure 1).

The beginning of deformation of the material of the lateral (peripheral) zone of the workpiece has a delay in relation to the deformation of the material of the central zone (Figure 1b). In its volume, the deformation patterns change during the process. In the initial period, the stress state of the material is characterized by uneven all-round compression. On the one hand, this is due to back pressure from the side walls of the working surface of the stamp at the place of formation of the flange part of the forging, on the other, it is from the side of the upset material of the central zone of the workpiece.

In the subsequent period of stamping, when the material flows out in the form of a technological burr, in the zone of the connector plane, the state of back pressure, as well as all-round compression, disappears. The deformed state of the material, as in the central zone, shortens in the direction of compression and elongates towards the center of the workpiece and the perimeter of the stamp. The maximum voltage of 239–256 MPa corresponds to the output of the material from the engraving along the connector plane.

The formation of the cylindrical section of the product occurs last of all under conditions of reverse extrusion, which is carried out due to counter radial displacements of the material of the central and peripheral zones of the workpiece (Figure 1c). Friction forces act on the side of the walls of the cylindrical section on the extruded material, which generally provides a scheme of uneven all-round compression. The intensity of stresses reaches values of 239–256 MPa.

**Figure 5:**
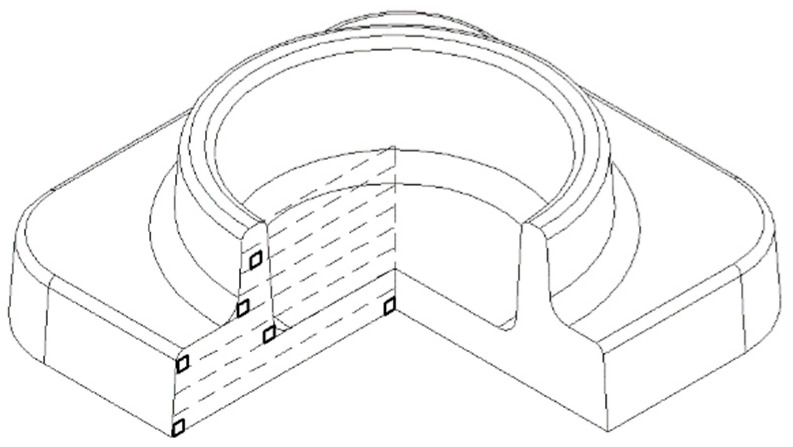
Diagram of the studied zones in the flange forging layers (numbering of eight layers from bottom to top).

The implementation of various mechanical deformation schemes during forging was expressed in the unevenness of the deformed state of the material in its volume and the change in shape and size of graphite inclusions. The location of the studied characteristic zones in the forging layers is shown in Figure 5.

**Figure 6:**
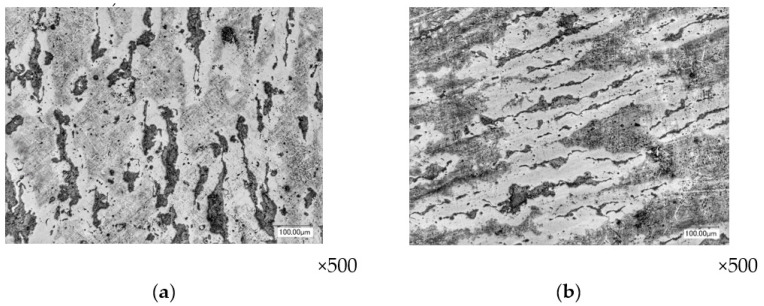
The shape of graphite inclusions in the side flange part of the forging (layer №1): layered section (**a**) and end section (**b**).

Along the perimeter of the flange part of the forging at the place where the material enters the burr groove, in the first layer, the deformation intensity has the value ε*_i_* = 2.02–2.52 and, in the second layer, it is ε*_i_* = 1.64–1.9. The deformed state of the material displays shortening in the direction of compression and elongation in two other directions, primarily to the perimeter of the engraving stamp. Graphite inclusions have an elongated shape in the direction of the flow of the material into the burr groove (Figure 6).

**Figure 7:**
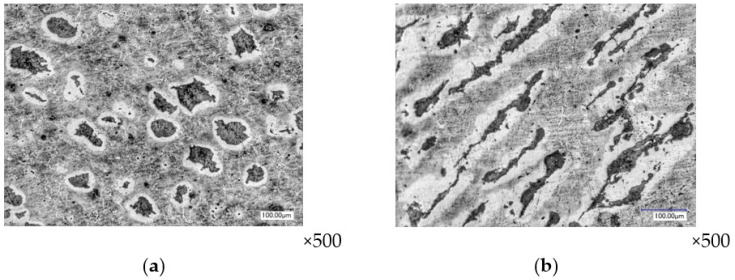
The shape of graphite inclusions in the central zone of the forging base (layer №1): layered section (**a**) and end section (**b**).

In the direction of the forging center, the deformed state of the first two layers decreases to the value of ε*_i_* = 1.41–1.52 and then increases again to ε*_i_* = 1.68–1.91. In the center, the deformed state of the material is characterized by shortening in the direction of compression and uniform elongation in the other two directions. Graphite inclusions have largely changed their spherical shape to a flattened lenticular shape, the position of which is perpendicular to the direction of precipitation (Figure 7).

**Figure 8:**
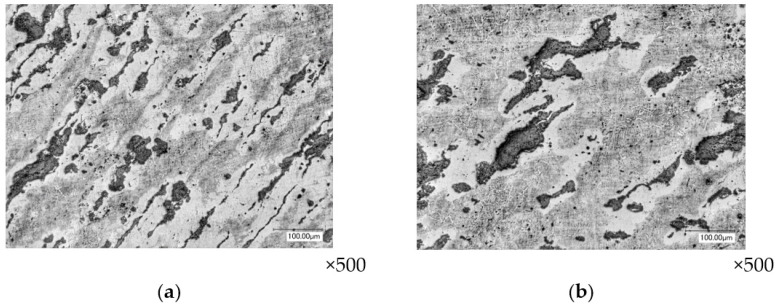
The shape of graphite inclusions on the radius section of the transition of the flange to the pipe part of the forging (layer №3): layered section (**a**) and end section (**b**).

In the third and fourth layers of the perimeter of the flange part of the forging, the deformation has the value ε*_i_* = 0.76–1.26, which is less than in the first two layers. As the transition to the pipe part of the forging occurs, the deformed state of these layers increases, reaching the value of ε*_i_* = 1.79–2.21 in the radius section. The deformed state of the material is characterized by elongation of its height and shortening in thickness. The change in the shape of graphite inclusions is also characterized by stretching in the direction of extrusion (Figure 8).

In the fifth and sixth layers of the pipe section, the deformed state continues to increase to the value ε*_i_* = 2.07–2.42. The highest value corresponds to the outer surface of the section, the smallest to the inner surface.

**Figure 9:**
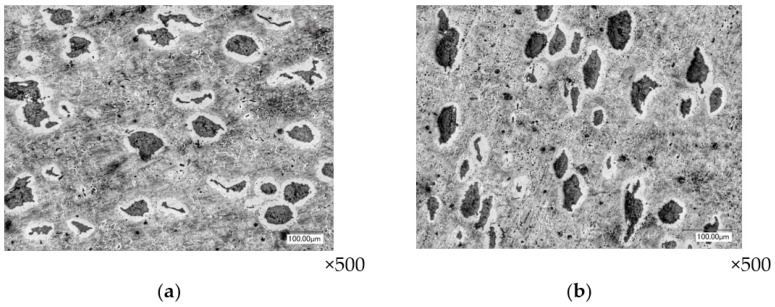
The shape of graphite inclusions in the end layers of the pipe forging section (layer №7): layered section (**a**) and end section (**b**).

In the seventh and eighth layers, the deformed state of the pipe section in comparison with the previous layers decreases: in the outer layers, to the value of ε*_i_* = 1.5, and in the inner layers, to the value of ε*_i_* = 1.35. Graphite inclusions have slightly lost their rounded shape (Figure 9).

Based on the result of the deformation analysis, it can be concluded that there is a significant unevenness of deformation in the forging volume, which is determined by the complex geometry of the deformation focus and, as a consequence, by different directions of the material flow [17]. The main flow occurs in the radial direction from the center of the workpiece to the burr groove. At the same time, it is impossible to fully agree with the position expressed by M.V. Storozhev, according to which, during the pre-stamping period, when the stamp figure is almost completely filled with metal, only the excess flows into the burr [19]. The results of mathematical modeling showed that during the pre-stamping period, due to the braking of the metal at the place of exit from the working area of the stamp, the direction of the main flow changes (bifurcates) and, additionally, by reverse extrusion, ensures the design of the cylindrical part of the product.

There were positive results of the stamping of the experimental batch of round forgings, in terms of the technological possibility of hot plastic deformation of ductile cast iron in open dies. In addition, the position that the deformability of the material is not a property, but the state of the material, was also confirmed and is determined by the conditions of deformation: speed, temperature, degree, and mechanical scheme of deformation. The most significant factor in increasing plasticity is considered to be deformation in a state of all-round compression. It can be created by applying special techniques that simulate hydrostatic pressure. In this work, it was created due to lateral pressure from the walls of the tool, carried out by optimizing the size of the initial workpiece. The diameter of the workpiece was selected in accordance with the dimensions of the inner contour of the flange part of the product. During deformation, the cast iron showed plasticity with a value of deformation intensity of ε*_i_* = 2.5.

The study of structural changes in cast iron with spherical graphite during deformation in open dies made it possible to evaluate the ability of graphite inclusions to undergo plastic deformation under conditions of complex loading and large degrees of deformation. During forging, the material underwent the greatest deformation in three zones: along the perimeter and center of the flange part and in the transition zone of the flange part to the cylindrical section. With almost identical degrees of deformation, ε*_i_* = 2.1–2.5, in all zones, graphite inclusions had different morphologies, which showed their dependence on loading conditions. The most difficult loading corresponds to the zone of the material’s exit from the die cavity into the burr groove, in which there is no state of all-round compression. Unlike the other two zones, in which the scheme of uneven all-round compression is implemented, on the one hand, by a deforming tool, and on the other, by adjacent layers of metal, there is no compressive stress in the considered zone from the material outlet side. The morphology of graphite inclusions is characterized by the greatest change in shape and size, including their fragmentation.

## 4. Conclusions

Based on the results of the work, the following conclusions were made:

1. By the method of hot volumetric stamping in an open die, an experimental batch of forgings of the round shape of the “flange” part, with satisfactory quality of the deformed metal in macro and microstructure, grinding, and orientation of the graphite phase in the direction of the main deformations, was obtained.
2. The results of the described work made it possible to determine the technological possibility of stamping forgings of round and similar shape in open dies in terms of creating conditions for uneven all-round compression of the material, while cast iron showed plasticity with a value of deformation intensity ε*_i_* = 2.5.
3. When stamping in open dies to create conditions for all-round compression, it is rational to use special techniques that simulate hydrostatic pressure, in particular, lateral pressure from the walls of the tool, carried out by selecting the size and shape of the initial workpiece.
4. The study of structural changes in ductile cast iron during deformation in open dies has shown the ability of graphite inclusions to undergo plastic deformation under conditions of complex loading with changes in shape and size, including their crushing.

## Figures and Tables

**Table 1:** Chemical composition of cast iron.

Content of the Elements, %							
C	Si	Mn	Ni	Cr	Mg	S	P
2.60	2.15	0.60	0.38	0.14	0.07	0.019	0.09

**Table 2:** Values of the intensity of deformation of cast iron in the studied flange zones.

	Sample №1				Sample №3				Sample №7		
point	area 1	area 2	area 3	area 4	area 1	area 2	area 3	area 4	area 1	area 2	area 3
1	2.03	2.16	1.41	1.91	0.70	1.46	1.61	2.12	2.28	1.60	1.71
2	2.02	1.38	1.38		0.95	1.37	1.63	1.79	2.38	1.87	2.51
3	2.43	1.28	1.60		1.00	1.50	1.64	2.21	2.38	1.54	1.57
4	2.53	1.42	1.62		1.20	1.54	1.77		2.29	1.58	1.36
5	2.26	1.45			1.22	1.58	2.28		2.02	1.59	1.63
6	2.51				1.25	1.62					
7	1.53				1.28	1.65					

**Table 3:** Stress intensity values (MPa) of cast iron in the studied flange zones.

	Sample №1				Sample №3				Sample №7		
point	area 1	area 2	area 3	area 4	area 1	area 2	area 3	area 4	area 1	area 2	area 3
1	239	238	192	189	187	199	195	204	144	143	148
2	235	194	180		190	203	193	196	144	144	144
3	251	186	188		188	200	189	200	140	145	156
4	255	198	183		197	195	193		145	145	151
5	239	199			194	192	199				
6	256				197	189					
7	205				201	195					

## Data Availability

The data presented in this study are available on request from the corresponding author.
